# Construction of a bundle for the safety of patients with mental disorders during hospitalization

**DOI:** 10.1590/0034-7167-2023-0263

**Published:** 2025-01-13

**Authors:** Marina Diva de Oliveira, Camila Souza de Almeida, Helen Cristiny Teodoro Couto Ribeiro, Richardson Miranda Machado, Juliano Teixeira Moraes

**Affiliations:** I Universidade Federal de São João del-Rei. Divinópolis, Minas Gerais, Brazil; II Universidade do Estado de Minas Gerais. Divinópolis, Minas Gerais, Brazil

**Keywords:** Patient Safety, Mental Health Recovery, Mental Disorders, Risk Management, Mental Health Assistance, Seguridad del Paciente, Recuperación de la Salud Mental, Trastornos Mentales, Gestión de Riesgos, Atención a la Salud Mental

## Abstract

**Objective:**

to develop a bundle for the safety of psychiatric patients during hospitalization.

**Methods:**

a methodological study conducted in two stages. In the first, a comprehensive literature review was developed through a scoping review and conducted to examine evidence on the safety of psychiatric patients during hospitalization. In the second, based on the evidence listed, a set of actions was developed for the safety of psychiatric patients during hospitalization.

**Results:**

twenty-six articles published between 2012 and 2022 were used, making it possible to categorize recommendations and build a bundle through four axes: safety culture; clinical decision-making; intervention planning; and interpersonal violence.

**Conclusions:**

the scientific evidence provided clear guidance on actions to improve the safety of psychiatric patients during hospitalization. This evidence also highlighted gaps in research, indicating the need for future studies in this area.

## RESUMEN

**Objetivo:**

construir un paquete para la seguridad de los pacientes psiquiátricos en el hospital.

**Métodos:**

estudio metodológico realizado en dos etapas. En primer lugar, se desarrolló una revisión exhaustiva de la literatura mediante una revisión de alcance y se llevó a cabo para examinar la evidencia sobre la seguridad de los pacientes psiquiátricos en el hospital. En el segundo, a partir de las evidencias enumeradas, se desarrolló un conjunto de acciones para la seguridad de los pacientes psiquiátricos durante la hospitalización.

**Resultados:**

se utilizaron 26 artículos publicados en el período entre 2012 y 2022, lo que permitió categorizar recomendaciones y construir un paquete en cuatro ejes: cultura de seguridad; toma de decisiones clínicas; planificación de intervenciones; y violencia interpersonal.

**Conclusiones:**

la evidencia científica proporcionó orientaciones claras sobre acciones para mejorar la seguridad del paciente psiquiátrico durante la hospitalización. Esta evidencia también puso de relieve las lagunas existentes en la investigación, lo que apunta a la necesidad de futuros estudios en esta área.

## INTRODUCTION

Safe practices in healthcare services are a topic of global relevance, and are the subject of important advances in public policies and evidence-based care practices. To this end, the assessment of service quality, improvement of patient safety and patient safety culture are structural components for achieving the effectiveness of this practice^(1)^.

Investment in risk management and the development of safe, quality care is well-known. However, when it comes to people with mental disorders, this investment does not reach the same level of progress seen in other specialty settings. Specifically, in hospital inpatient services, unique challenges related to the safe care of inpatient psychiatric patients are identified, highlighting the urgency of investing in research, in addition to developing policies and applying them to the clinic^(2)^.

The challenge of promoting safe mental healthcare is even more relevant when characterizing its specificity in clinical management. It is important to note that patients with mental disorders go through the Psychosocial Care Network not only due to demands related to the occurrence of distinct events and incidents, such as self-harm, violence and suicide, but also due to demands related to routine care needs. Such events and incidents, associated with other risks common to patients in other specialties, indicate the relevance and scope of this topic. Therefore, it is necessary to invite the expansion of the scope of studies regarding patient safety and discussion about safe care for patients with mental disorders^(3)^.

The World Health Organization initiative, called “Right is Quality”, proposes good practices in mental health that ensure patients can exercise their rights as citizens. The proposed approaches involve ensuring the quality of services, practices and patient safety through a focus on recovery. Recovery represents an approach that aims not only to restore health, but also to strengthen and empower people who experience experiences resulting from mental disorders^(4)^.

Considering the risks to which psychiatric patients are vulnerable, and specifically in hospital settings, studies indicate a greater number of adverse events present in non-psychiatric hospitalizations of patients with mental disorders than in those of the general population. Daumit *et al*.^(5)^ state that, during medical and surgical hospitalizations, people with schizophrenia, for instance, were at least twice as likely to suffer serious adverse events when compared to people without schizophrenia who suffered from the same health problem. Furthermore, according to the authors, the events were associated with poor clinical outcomes during hospitalization, which increased length of hospitalization, costs, use of Intensive Care Unit and hospital death for patients with schizophrenia, warning of the economic and social impacts associated with the issue^(6)^.

It is known that a significant number of people suffer from some type of mental disorder. Globally, estimates indicate that 4.4% of people worldwide suffer from depressive disorders and 3.6% from anxiety disorders, indicating a sustained trend of growth in these conditions^(7)^. In addition to the direct adverse effects that mental disorders can produce, there is evidence that mental disorders are associated with an increase in the frequency and severity of other chronic diseases, excessive disabilities and increased absenteeism at work. This evidence highlights the need for attention to this issue.

Recognizing the gaps in research in mental health and, specifically, related to the safety of patients with mental disorders during hospitalization, correlated to aspects of vulnerability highlighted, as well as advances and limits of follow-up, this study proposes to build a bundle based on scientific evidence on the safety of patients with mental disorders in hospitals, starting from the following question: what scientific evidence in the context of hospitalization is available on the safety of patients with mental disorders?

## OBJECTIVE

To develop a safety bundle for patients with mental disorders in hospital.

## METHODS

### Ethical aspects

The research project was analyzed and approved by the Research Ethics Committee, in compliance with the ethical principles required by Resolution 510/2016 of the Brazilian National Health Council. Consent was waived, as this was a scoping review and did not involve the use of biological materials collected and stored as part of institutional routines, without adding risk to research participants or harming their well-being.

### Study design, period and location

This is a methodological study carried out in two stages. The first consisted of conducting a scoping review to map scientific evidence on the safety of patients with mental disorders in hospitalization. The second stage consisted of constructing a bundle based on the knowledge production mapped by the review. Thus, the bundle was composed of items on the safety of psychiatric patients in hospitalization, based on the evidence recommended by the identified studies.

The scoping review was conducted in accordance with the JBI proposal, and was based on the following research question: what scientific evidence is available in the context of hospitalization on the safety of patients with mental disorders? This method suggests five stages: 1. Research question identification; 2. Relevant study identification; 3. Study selection; 4. Data analysis; and 5. Data grouping, synthesis and presentation^(8,9)^.

### Study protocol

A research protocol was developed prior to the review to provide transparency to the process. Therefore, this scoping review had its research protocol registered in the Open Science Framework, through the registry available at: https://doi.org/10.17605/OSF.IO/2RMZT .

To construct the research question, the Population, Concept and Context (PCC) strategy was used for a scoping review. The strategy was defined as follows: P (Population) - patients with mental disorders; C (Concept) - patient safety with mental disorders; and C (Context) - hospital admission. Based on these definitions, the guiding question was established: what scientific evidence is available in the context of hospital admission on patient safety with mental disorders?

To identify the descriptors and keywords, an initial search was conducted on the PubMed portal and the DeCS/MeSH database to identify the main descriptors and keywords used in studies addressing the topic of interest based on the combination of the MeSH identified for the research mnemonic. Subsequently, the search strategy defined was: (Hospitalization OR Hospitalizations) AND (“Patient Safety” OR “Patient Safeties” OR “Safeties, Patient” OR “Safety, Patient”) AND (“Mental Health” OR “Health, Mental” OR “Mental Disorders” OR “Mental Disorder” OR “Psychiatric Illness” OR “Psychiatric Illnesses” OR “Psychiatric Diseases” OR “Psychiatric Disease” OR “Mental Illness” OR “Illness, Mental” OR “Mental Illnesses” OR “Psychiatric Disorders” OR “Psychiatric Disorder” OR “Behavior Disorders” OR “Diagnosis, Psychiatric” OR “Psychiatric Diagnosis” OR “Mentally Ill Persons” OR “Mentally Ill Person” OR “Person, Mentally Ill” OR “Persons, Mentally Ill” OR “Mentally Ill” OR “Ill, Mentally” OR “Mental Patients”).

Study selection took place between July and September 2022 in seven databases, as follows: 1. PubMed/MEDLINE; 2. Latin American and Caribbean Literature on Health Sciences (LILACS); 3. Embase; 4. Cochrane; 5. CINAHL; 6. Web of Science; and 7. Scopus. Moreover, a free search was carried out on Google^®^ and in theses and dissertations on the subject, as they provided sufficient elements to compose the review and answer the research question.

Scientific research studies published in full in English, Portuguese and Spanish up to 2021, addressing the safety of patients with mental disorders during hospitalization, were included. Research protocols, editorials, reviews and letters were excluded. Studies were screened using Rayyan^®^, a free application available on the web, used to assist in review and meta-analysis type research, which allows for faster initial screening for reading titles and abstracts. In case of doubts about the relevance of a study based on abstracts, the full article was retrieved, as was the case for all other studies that met the inclusion criteria. Studies that were not aligned with the objective and the established review question were excluded. The reading was performed by two independent reviewers, and disagreements were resolved by a third reviewer, reaching a consensus.

### Analysis of results

Initially, the title and abstract of all identified studies were assessed, after which the selected studies were read in full based on the inclusion criteria described. The data extracted from studies were pre-established in the research protocol. For mapping, a structured instrument was used with information corresponding to study design, year of publication, country of origin, objective, type of research, population, location, description of care for the safety of patients with mental disorders in hospitalization and their results. These data were entered into a spreadsheet created in Microsoft Excel 2010^®^.

The methodological level of evidence (LoE) of each study was added to these data. To this end, the level of evidence of studies was classified using the classification system recommended by JBI, as follows: level 5 (expert opinion); level 4 (descriptive observational studies); level 3 (analytical observational studies); level 2 (quasi-experimental studies); and level 1 (experimental studies). The data were presented descriptively (number of articles) with relevant literature. The data from studies were summarized, grouped, and presented in charts.

In the second stage, the bundle was constructed using the evidence categorized in the scoping review. The bundle was structured into four categories, categorized by similarity of content, in intervention planning, safety culture, interpersonal violence and clinical decision-making.

## RESULTS

**Figure 1:**
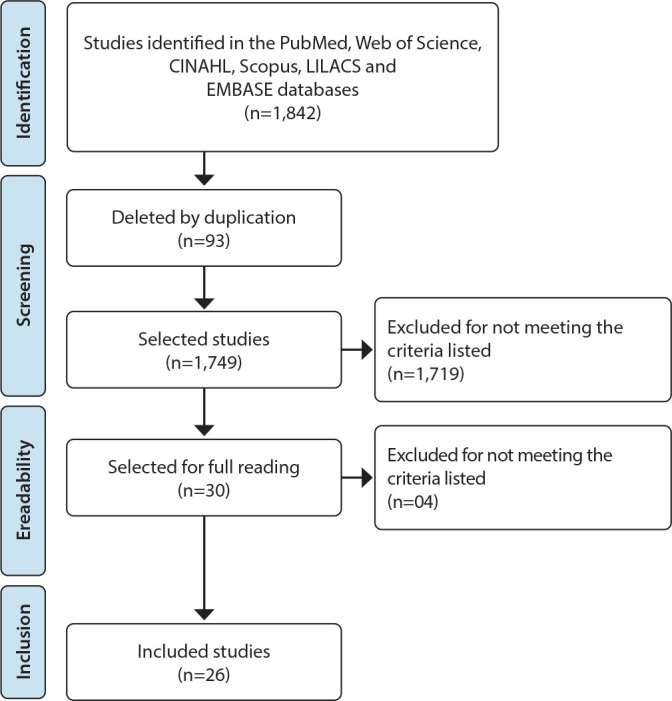
Study selection process flowchart, 2023

A total of 1,842 studies were identified; of these, 93 were excluded due to duplication. After initial screening of titles and abstracts, 30 articles were included for full reading. After analysis, the final sample consisted of 26 studies. The specificity of the topic did not allow finding gray literature that answered the research question. The study selection process is represented in the Preferred Reporting Items for Systematic Reviews and Meta-Analyses (PRISMA) flowchart (Figure 1).

Of the included studies, 85% (n=22) of articles were available in English and were published between 2012 and 2021.

The studies come from countries with greater consolidation of patient safety policies, such as the USA, and mental health policies, such as Brazil, and represent places where innovative care practices are being established.

Brazil is ranked 3^rd^ in the publication rankings and is the only country in Latin America with studies in this area. Brazilian studies covered the areas of psychiatric risk assessment, adverse events in psychiatric hospitalization, understanding of patient safety by general hospital staff, and implementation of risk management for patient safety.

Given the breadth of topics covered in the studies found, it was necessary to stratify the results into thematic categories. Analyzing the most relevant findings and results of studies, categorization was carried out into broad thematic areas and subcategories, such as safety culture, clinical decision-making, intervention planning and interpersonal violence, presented in Chart 1.

**Chart 1:** Categorization of the topics covered in the articles included in the study. Minas Gerais, Brazil, 2023

Category	Subcategories
Safety culture	Medication error; safe practices in medication administration; quality improvement; sexual safety; risk management; adverse events; night nursing observations; physical environment safety; assertive and active communication; risk of falling; harmful behavior: self-harm, suicidal behavior, and self-neglect (A2, A4, A5, A6, A7, A8, A9, A10, A11, A14, A15, A18, A19, A21, A23, A24, A25, A26)^(3,7,10-25)^
Clinical decision-making	Continuing care after discharge; discharge planning; planned care in hospitalization of patients with severe mental disorders; psychiatric risk assessment checklist and routine (A13, A20, A22)^(26-28)^
Intervention planning	Micropolitics of care for psychiatric inpatients (A3, A16, A17)^(2,29-31)^
Interpersonal violence	Approach related to concerns about episodes of violence by patients and staff (A1, A12)^(31,32)^

The “safety culture” category accounted for 69% (n=18) of studies, with a variety of subcategorizations, which correlate with the main thematic axes addressed in the policies and guidelines on “patient safety”. The category related to “clinical decision-making” accounted for 12% of studies (n=3) as well as the category “intervention planning” (n=3). The category “interpersonal violence” accounted for 8% of studies (n=2) (Chart 1).

Using this evidence, a safety bundle for psychiatric patients during hospitalization was developed (supplementary material).

Due to the findings and the complexity of the axes, a multiaxial bundle was developed that aligns a set of bundles in correspondence with the categories and their respective interventions. It was established through four axes: safety culture; clinical decision-making; intervention planning; and interpersonal violence. All were aligned with international standards of the recovery approach through safety and quality as human rights.

## DISCUSSION

As noted, the studies addressed strategies that can be considered relevant for the safe care of psychiatric patients during hospitalization. Thus, a bundle was created with the main recommendations on the safety of psychiatric patients during hospitalization. The topics addressed were based on the bias of care production and strategies focused on rights as quality.

The evidence stratified by categories made it possible to list recommendations such as: assessing patients; identifying the risk; creating an organizational and structural culture; raising awareness among employees; organizing the work process both during hospitalization and during safe and referenced discharge; identifying signs of worsening and triggers for worsening; safely administering medication; minimizing the risk of falling; preventing suicide and episodes of heteroaggression.

The studies categorized in patient safety culture mention the investigated treatment and care processes in hospitalization of patients with mental disorders.

Within patient safety culture, some segments must be highlighted and understood to develop strategies that strengthen the patient safety policy for psychiatric patients within healthcare institutions.

As for adverse events in psychiatric hospitalization, it is important to understand that adverse events are at least partially the result of omission or provision of clinical care. They can result from psychiatric illnesses and are influenced by many patient, clinical and social factors. Psychiatric patients have a high risk of suffering adverse events, taking into account the association between therapeutic specificities and the symptomatology of psychopathologies that sometimes lead patients to behaviors such as aggression, isolation, suicide, escape and reduced self-defense^(10,23)^.

Decision-making in clinical practice stands out, such as the exercise of action based on extraction of evidence, interpretation and management of risks, integration of a care plan with patients, in addition to focusing on the health-disease process and review of actions and results to improve patients’ health condition and recovery. Moreover, the development of clinical judgments and decisions related to incident management, risk assessment, diagnosis and discharge management are decisive topics of publication in the studies analyzed.

In this category, hospital discharge management is an important stage to be considered for the care of psychiatric patients, given the relationship with the occurrence of readmissions and the connection between mental disorders and chronic diseases. This is a process that begins at hospitalization so that patients can be mapped in their entire context to identify vulnerabilities and weaknesses. From this, a care network is built in their territory. Therefore, it is recognized that, for a safe discharge, resources and care planning must be carried out throughout the entire period of hospitalization, in addition to involving post-hospital discharge care, ensuring humane treatment and recovery^(33)^.

In the discharge management process, the role of nurses stood out. Knowledge of discharge documents, guidance on continuity of treatment in a clear and effective manner, recognition of possible clinical limitations and patients’ understanding of treatment, clinical condition and signs of worsening, and the involvement of family/guardian and community must occur and clarify all doubts at hospital discharge^(33)^. This process can influence the reduction of readmission, improvement of well-being, increase of adherence to treatment, mainly highlighting the relationship with suicide reduction.

The studies mainly related the prevalence, management and prevention of violent/aggressive behaviors. Patient aggression in mental health settings is a risk of greater relevance related to psychiatric patients, due to the symptoms of some mental disorders. It presents an ongoing challenge for organizations and healthcare professionals, given the peculiarities of management as well as the occurrence of physical and psychological trauma to other patients, employees and visitors^(31)^.

In this regard, it is highlighted that aggressive signals can reduce aggression, i.e., there is a possibility of preventing aggression through anticipation of violence if it occurs based on clinical management corresponding to identified risks.

Safe care also comes from assistance and safety culture, consolidated by the attitudes, behaviors and values of professionals and organizations. When developing patient safety, it is important to remember the interrelational diversity of the concept so that all areas are considered in the development work^(30)^. Investment in research, policy development and translation of these into clinical practice is necessary.

The consolidation of strategies that consider the local reality of services, identifying organizational weaknesses and also those of professionals, makes it possible to develop more assertive macro and micro interventions.

The micropolitics of mental health work was presented as a space for engagement with the uniqueness and weaknesses of patients, recognizing the extrinsic and intrinsic challenges and the complexity of caring for psychiatric patients in hospitalization. It is assumed that patient safety will occur through the organization and exercise of joint practices.

It reveals work established with the meeting between care production, management and training, proposed as an analytical and problematizing process of established practices and lines of connection.

The results demonstrate initiatives for safe care for psychiatric patients, albeit timidly. It is known that there is historical neglect regarding mental health.

Although the 26 pieces of evidence found support the effectiveness of many psychiatric patient safety interventions in hospital settings, there is a notable lack of research that addresses broad cultural, social and economic issues that truly affect safe care as a recovery practice and specific interventions that can address them.

Therefore, this study reinforces the existence of gaps in mental health research on the safety of psychiatric patients in hospitalization.

However, the studies presented direct safe care through evidence-based interventions that have proven effective. They can be recognized as a menu of action options anchored in patient safety in hospital services, revealing possible paths for continuous improvement and effective consolidation of quality culture. Thus, this bundle on patient safety for mental disorders in hospitalization, as a systematized care tool, validates the importance of critical thinking about the necessary safe care, especially in a highly vulnerable environment.

Although there are challenges in implementing this approach, the examples of evidence presented and listed here, through the bundle recommendations, concretely reveal what can be done and in which areas of research we should invest.

### Study limitations

Although the research has made it possible to generate knowledge, many studies that may address patient safety care do not always use this term in their descriptors and therefore may not have been included in the study.

### Contributions to nursing, health or public policy

In the context of public patient safety policies, although significant advances have been made, there is still much work to be done, and nursing plays a prominent role in this area. Thus, this study contributes to the connection between knowledge and practice, with the aim of enabling nursing to intertwine knowledge and practices in safe, humane and ethical care in mental healthcare.

## CONCLUSIONS

Based on the recommendations highlighted by the scoping review, it was possible to develop a bundle to ensure the safety of psychiatric patients in hospital settings.

All studies address care designed with governance to prevent risks and consolidate the safety of psychiatric patients during hospitalization. The scoping review reports on evidence in the field of psychiatric patient safety in hospitalization settings, addressing the research question.

In the bundle, it was possible to categorize four axes that present the focus of care: safety culture; clinical decision-making; intervention planning; and interpersonal violence. All were aligned with international standards of the recovery approach through safety and quality as human rights.

This instrument, constructed through a synthesis of evidence, may contribute to future research, programs and policies by providing researchers with a consensus on the recommendations for safe care identified by current studies. In addition, it may be possible to identify research priorities related to the topic.

Finally, the complexity of mental healthcare and the opportunity to highlight the relevance of patient safety during hospitalization in consolidation of care with a focus on recovery were highlighted.

## Supplementary Material

0034-7167-reben-78-01-20230263-suppl01

## Data Availability

http://dx.doi.org/10.17632/nvn7byzf7z.1
