# Supplementary material for: Construction of a bundle for the safety of patients with mental disorders during hospitalization
Source: Rev Bras Enferm. 2025 Jan 13;78(1):20230263. doi: 10.1590/0034-7167-2023-0263 (PMC11731854; doi:10.1590/0034-7167-2023-0263)
Supplement: 0034-7167-reben-78-01-20230263-suppl01 [file 0034-7167-reben-78-01-20230263-suppl01.pdf]

Construção de um *bundle* para a segurança do paciente com transtornos mentais em internação hospitalar

Material suplementar

Quadro 1 - Estudos analisados segundo título, ano, país, objetivo, tipo de estudo e principais resultados, Minas Gerais, Brasil, 2023.

nº	NE*	Título, país, ano/publicação	Objetivo	Tipo de estudo	Principais resultados
1	NE=5	A data science approach to predicting patient aggressive events in a psychiatric hospital[1]<br><br>Estados Unidos (2018)	Capitalizar a extensa quantidade de dados disponíveis em registros eletrônicos de saúde para prever eventos agressivos de pacientes	Estudo retrospectivo	Os preditores de eventos agressivos de pacientes incluíram falta de moradia, ter sido condenado por agressão e ter testemunhado abuso. O algoritmo também foi usado para gerar um limite de probabilidade de custo otimizado para um evento agressivo. Sugere cobertura individualizada de equipe para pacientes com maior risco de agressão
2	NE=4	An assessment of levels of safety in psychiatric units[2]<br><br>Estados Unidos (2017)	Compreender os padrões de incidentes em relação aos diferentes tipos de espaços dentro de uma unidade psiquiátrica	Estudo de método misto	O suicídio é mais comum em quartos de pacientes e banheiros, e a violência é mais frequente em quartos de dia. Os resultados dos grupos focais produziram recomendações perspicazes
3	NE=3	An emergency department intervention and follow-up to reduce suicide risk in the VA: Acceptability and effectiveness[3]<br><br>Estados Unidos (2016)	Avaliar a aceitabilidade e a utilidade percebida da intervenção de Planejamento de Segurança com acompanhamento e monitoramento por telefone para indivíduos suicidas que não necessitaram de internação	Estudo de intervenção Multicêntrico	A maioria dos participantes considerou a intervenção SAFE VET útil para mitigar o risco de suicídio e em aumentar a probabilidade de comparecer a consultas de acompanhamento de saúde mental

4	NE=4	A new paradigm for mental-health quality and safety: are we ready?[4]<br><br>Austrália (2019)	Desenvolvimento de um Quadro de Qualidade e Segurança em Saúde Mental com áreas prioritárias co-desenhadas para melhoria	Estudo com abordagem qualitativa	Proposta deum quadro com a descrição do mapa dos recursos/competências exigidos pelos serviços de saúde mental para incorporar a melhoria contínua da qualidade
5	NE=4	Application of the SEIPS model to analyze medication safety in a crisis residential center[5]<br><br>Estados Unidos (2018)	Analisar os fatores críticos relacionados à estrutura e aos processos do sistema de trabalho em saúde mental que ameaçam as práticas seguras de administração de medicamentos	Estudo com Abordagem qualitativa	Interrupções durante o processo de medicação, necessidades de treinamento em farmacologia e processos de documentação como as principais oportunidades de melhoria
6	NE=4	A qualitative understanding of patient falls in inpatient mental health units[6]<br><br>Estados Unidos (2014)	Determinar recomendações específicas do segmento de mercado para "vender" prevenção de quedas em psiquiatria hospitalar aguda	Estudo descritivo	Os achados foram agrupados em avaliação de risco de queda, precauções clínicas de risco de queda, prevenção de quedas programática e prevenção de quedas em psiquiatria
7	NE=4	Communication elements supporting patient safety in psychiatric inpatient care[7]<br><br>Finlândia (2015)	Descrever quais elementos da comunicação subsidiam a segurança do paciente em internação psiquiátrica sob a ótica da equipe de enfermagem	Estudo com abordagem qualitativa	Identificar os pacientes, incluindo os riscos e definindo os cuidados motiva o trabalho multidisciplinar para manter os pacientes seguros
8	NE=4	Defining patient safety events in inpatient psychiatry[8]<br><br>Estados Unidos (2021)	Descrever a segurança do paciente em internação psiquiátrica, enquadrada por uma aplicação da estrutura de segurança do paciente do Institute of Medicine	Estudo com abordagem qualitativa	Caracterização de uma listagem de definição de Eventos de segurança do paciente
9	NE=4	Development and pilot implementation of a	Desenvolver e implementar um protocolo de busca para melhorar	Estudo qualitativo	Estabelecido protocolo que contou com um cartaz usando um auxílio mnemônico para conscientizar

		search protocol to improve patient safety on a psychiatric inpatient unit[9]<br><br>Canadá (2017)	a segurança de pacientes, funcionários e visitantes, evitando que itens inseguros entrassem em uma unidade de internação trancada		pacientes, funcionários e visitantes sobre quais itens não poderiam ser trazidos para a unidade e sessões educativas sobre o protocolo de busca foram fornecidas. A diferença entre o número de incidentes antes e após a implementação do protocolo de busca foi estatisticamente significativa
10	NE=4	Development of psychiatric risk evaluation checklist and routine for nurses in a general hospital: ethnographic qualitative study[10]<br><br>Brasil (2015)	Desenvolver uma lista de verificação de avaliação de risco psiquiátrico e rotina para enfermeiros, a Lista de Verificação de Avaliação de Risco Psiquiátrico, como modelo alternativo para identificação precoce e manejo dessas situações em hospitais gerais	Estudo etnográfico	Desenvolvido um modelo para detectar e intervir precocemente em atuações comportamentais de pacientes psiquiátricos através de checklist. Foi considerado a forma como os profissionais descrevem e/ou relatam esses comportamentos, e por meio de uma rotina inserida em sua prática diária
11	NE=4	Adverse events in a psychiatric hospitalization unit[11]<br><br>Brasil (2022)	Descrever os eventos adversos presentes na internação psiquiátrica, analisando-os à luz da teoria do erro humano	Pesquisa exploratória, qualitativa	Evidenciaram-se eventos adversos medicamentosos por erros de administração ou por reações adversas a medicamentos, que produzem danos como impregnação, reações extrapiramidais associadas aos riscos de queda e broncoaspiração pela sonolência e/ou sedação. Outros danos relacionam-se à agressividade do paciente, que produz lesões corporais a si ou a outro, como durante uma tentativa de suicídio ou uso de violência como comportamento de fuga ou defesa
12	NE=4	Examination of the effectiveness of the Mental Health Environment of Care Checklist in reducing suicide on inpatient mental health units[12]<br><br>Estados Unidos (2012)	Avaliar o efeito da identificação e redução de riscos em suicídios de pacientes internados no Veterans Health Administration	Estudo qualitativo	A implementação do Mental Health Environment of Care Checklist foi associada a uma redução na taxa de suicídio de pacientes internados em hospitais Veterans Health Administration nacionalmente

13	NE=1	Interventions to improve discharge from acute adult mental health inpatient care to the community: systematic review and narrative synthesis[13]<br><br>Reino Unido (2019)	Identificar a base de evidências para intervenções para apoiar a continuidade dos cuidados e a segurança na transição da internação de saúde mental aguda para serviços comunitários no momento da alta	Revisão Sistemática	As intervenções seguiram uma abordagem nomeada distinta (ou seja, Intervenção em Tempo Crítico, Modelo de Alta Transitória), outras foram agrupadas com base em componentes-chave (ou seja, apoio de pares, envolvimento do farmacêutico). Os principais problemas que as intervenções procuraram abordar foram reduzir a readmissão, melhorar o bem-estar, reduzir a falta de moradia, melhorar a adesão ao tratamento, acelerar a alta e reduzir o suicídio
14	NE=3	Mitigating risk in Norwegian psychiatric care: Identifying triggers of adverse events through Global Trigger Tool for psychiatric care[14]<br><br>Noruega (2019)	Relatar os achados de gatilhos associados a eventos adversos identificados por uma versão do Global Trigger Tool - Psychiatry adaptado para tratamento psiquiátrico de base hospitalar norueguês	Estudo Retrospectivo	Existe uma relação clara entre a presença de gatilhos e eventos adversos
15	NE=4	Patient safety in a general hospital's psychiatric hospitalization unit: a phenomenological study[15]<br><br>Brasil (2021)	Compreender o significado da segurança do paciente para uma equipe multiprofissional em uma unidade psiquiátrica de um hospital geral	Estudo fenomenológico	O significado de segurança do paciente psiquiátrico foi entendido como abarcando experiências de gestão de equipe que enfatizam a coerção física e o controle da sintomatologia ao mesmo tempo em que indicam a expectativa de elaboração de novos procedimentos que contemplem a humanização. Inclui também questões de composição organizacional e dificuldades vivenciadas em relação à estrutura física, sua interferência no processo de prestação de cuidados seguros e expectativas de melhoria
16	NE=1	Patient safety in inpatient mental health settings: a systematic review[16]<br><br>Reino Unido (2019)	Identificar e sintetizar a literatura sobre segurança do paciente em ambientes de internação de saúde mental	Revisão sistemática e metassíntese	Foram identificadas dez categorias de pesquisa: violência interpessoal, intervenções coercitivas, cultura de segurança, dano a si mesmo, segurança do ambiente físico, segurança da medicação, afastamento não autorizado, tomada de decisão clínica, prevenção e controle de quedas e infecções

17	NE=4	Patient safety in psychiatric inpatient care: a literature review[17]<br><br>Finlândia (2013)	Compreender o conceito de segurança do paciente e sua intenção na internação psiquiátrica, e identificar fatores na gestão da organização, nos papéis da equipe e dos pacientes que constituem a segurança do paciente	Revisão de literatura	A gestão da organização tem o papel principal na segurança do paciente dentro da cultura da organização por meio de liderança, práticas de segurança e criação de boas condições e ambiente de trabalho para os funcionários
18	NE=3	Reducing adverse medication events in mental health: Australian National Survey[18]<br><br>Austrália (2020)	Determinar até que ponto as práticas de segurança de medicamentos baseadas em evidências foram implementadas em unidades de internação de saúde mental públicas e privadas em toda a Austrália	Estudo retrospectivo e transversal	As lacunas nas práticas de segurança de medicamentos incluíram o uso limitado de sistemas de fornecimento de pacientes individuais para distribuição de medicamentos, uma alta dependência de sistemas de estoque de enfermaria e alta dependência de sistemas baseados em papel para prescrição e administração de medicamentos. A prestação de serviços, o envolvimento do farmacêutico clínico em serviços de reconciliação médica, monitoramento de medicamentos terapêuticos e rondas interdisciplinares deve ser aumentado. Os serviços de alta e pós-alta foram as principais lacunas na prestação de serviços
19	NE=3	Retrospective analysis of reported suicide deaths and attempts on veterans health administration campuses and inpatient units[19]<br><br>Estados Unidos (2021)	Revisar relatórios de análise de causa raiz de eventos de suicídio por unidade hospitalar para fornecer recomendações de prevenção de suicídio para cada área	Estudo retrospectivo	O enforcamento foi responsável por 71% dos óbitos nas unidades de saúde mental e 50% das mortes nas unidades médicas. A superdosagem foi responsável por 55% dos óbitos e 68% das tentativas em unidades residenciais e o único método que resultou em óbito nos serviços de emergência. Nos centros comunitários, enforcamento, overdose e asfixia foram responsáveis por 64% das mortes. Os tiros foram responsáveis por 59% das mortes em áreas hospitalares e 100% das mortes em áreas clínicas

20	NE=1	Safety of service users with severe mental illness receiving inpatient care on medical and surgical wards: A systematic review[20]<br><br>Austrália (2018)	Sintetizar as evidências sobre a probabilidade de danos e mortalidade em enfermarias de internação médica e cirúrgica para pessoas com doença mental grave	Revisão Sistemática	Houve evidências de que os eventos adversos são maiores em pessoas com transtornos mentais. Uma maior probabilidade de emergência em vez de cuidados planejados e menor acesso ao tratamento foram identificados como potenciais fatores contribuintes para esses eventos adversos. Além disso, os usuários do serviço com transtornos mentais foram mais propensos a ter maior tempo de permanência, associado a um maior custo do atendimento. A gravidade da doença mental aumentou a probabilidade de dano ou morte, e as pessoas com esquizofrenia eram mais propensas do que as pessoas com outras doenças mentais a experimentar esses resultados adversos. Há evidências de que pessoas com transtorno mental recebem cuidados de saúde de menor qualidade. Cuidados de maior qualidade e melhor planejados são necessários para superar as desigualdades de acesso
21	NE=4	Safety of patients with mental disorders: a collective construction of strategies[21]<br><br>Brasil (2020)	Descrever a implantação da gestão de risco para a segurança do paciente com transtorno mental por meio da pesquisa-ação	Pesquisa-ação	Foram desenvolvidas três estratégias consideradas como tecnologias gerenciais para o doente mental: diagrama de gerenciamento de risco para segurança do paciente; protocolos de segurança do paciente; e proposta textual de software para gestão interna de notificações de incidentes
22	NE=3	Safety of reassessment-and-release practice for mental health patients boarded in the emergency department[22]<br><br>Estados Unidos (2018)	Comparar a mortalidade e a utilização de serviços de saúde por disposição de alta do pronto-socorro e status de solicitação de leito de internação	Um estudo de coorte retrospectivo	Uma visita ao pronto socorro dentro de 12 meses foi significativamente maior entre os pacientes que receberam alta sem iniciar tratamento direcionado do que aqueles iniciaram tratamento e que receberam alta ou internados

23	NE=4	Suicide-specific safety in the inpatient psychiatric unit[23]<br><br>Estados Unidos (2015)	Ilustrar a operacionalização de uma cultura de segurança específica ao suicídio	Estudo qualitativo	A prevenção do suicídio é essencialmente a redução do risco de suicídio e a manutenção dessa redução. A prevenção da autoagressão é proposta através da redução o acesso a meios letais, conectando e colaborando com o paciente, utilizando as melhores práticas, reduzindo os sintomas agudos, ajudando os pacientes a desenvolver habilidades saudáveis de enfrentamento e resolução de problemas , aumentando a esperança, identificando razões para viver, fortalecendo as conexões interpessoais e tratando os pacientes com o máximo de cuidado compassivo e respeito
24	NE=4	The psychiatric ward environment and nursing observations at night: A qualitative study[24]<br><br>Reino Unido (2020)	Compreender a experiência vivida de ser um paciente internado em enfermaria psiquiátrica à noite com foco em observações intermitentes e contribuir para o desenvolvimento de uma ferramenta para monitorar o ambiente da enfermaria psiquiátrica para melhoria da qualidade	Pesquisa qualitativa	Distúrbios ambientais, incluindo luz e ruído, invasão de privacidade e considerações de segurança na enfermaria, contribuíram para o distúrbio do sono. As consequências não intencionais de distúrbios causados por observações noturnas intermitentes, e pela equipe em geral e outros pacientes formaram o cerne dos pacientes internados
25	NE=4	What causes medication administration errors in a mental health hospital? A qualitative study with nursing staff[25]<br><br>Reino Unido (2018)	Investigar as causas de erros de administração de medicamentos que afetam pacientes internados em um hospital de saúde mental do National Health Service no noroeste da Inglaterra	Pesquisa qualitativa	A maioria dos erros foram identificados tendo como influente relação com os níveis de pessoal inadequados, combinação desequilibrada de habilidades de pessoal, interrupções/distrações, preocupações com a forma como a tarefa de administração de medicamentos foi abordada e problemas de comunicação

26	NE=4	Sexual Safety for In-Patient Mental Health Care-The Democratic Diagnosis of Change[26]<br><br>Reino Unido (2019)	Reduzir o risco de violência sexual em ambientes de internação de saúde mental	Pesquisa qualitativa	Oferece uma conceituação de segurança sexual de pacientes internados em um contexto de saúde mental

Nota: *NE: Nível de evidência.

Quadro 2 – *Bundle* Segurança do paciente psiquiátrico em Internação Hospitalar, Minas Gerais, Brasil, 2023.

Categoria 1	Intervenções	Ações
**TOMADA DE DECISÃO CLÍNICA**	**Promoção de cuidados integrados e continuados após alta**	Garantir uma internação de curta permanência e com fortalecimento da contrareferência para atenção primária
		Elencar um(a) coordenador(a) de alta para promover a transferência de cuidados e comunicação entre os cuidados primários e secundários
		Promover o gerenciamento de sintomas, adesão à medicação e suporte familiar aprimorado
		Estabelecer programa de alta pela equipe de enfermagem - medicação hospitalar direta, monitoramento telefônico de acompanhamento
		Promover grupo de reentrada (após alta) com reuniões de grupo de curta duração com abordagem psicoeducacionais
		Executar um programa de alta hospitalar (pré alta) em 6 etapas aos cuidadores. Com foco em parceria terapêutica integrada, educação em saúde mental e serviços orientados para as necessidades.
	**Manejo da urgência psiquiátrica**	Determinar um protocolo de manejo em urgência psiquiátrica - oferta de leito crise em pronto atendimento com recursos ambulatoriais para acomodar pacientes com necessidades psiquiátricas contínuas e com reavaliação no dia seguinte
**Categoria 2**	**Intervenções**	**Ações**
	**Prevenção de quedas**	Definir campanhas para promoção de marketing social aos profissionais gerais, pacientes e familiares
		Definir um modelo de avaliação de risco de queda que combine intervenções com os fatores de risco
		Desenvolver um plano de cuidados individualizado e multifatorial a partir da avaliação de risco de queda
		Identificar fator de risco de queda: 1. uso de polifarmácia (hipnóticos, sedativos), 2. perda de equilíbrio corporal, 3. hipotensão postural, 4. presença de escada no pátio, 5. incontinência urinária, 6. presença de lâmpadas queimadas na unidade e higienização do piso em momento de trânsito dos pacientes. 7. queda recente; 8. fraqueza muscular, 9. distúrbio comportamental, agitação ou confusão, 10. aqueles >85anos
	**Prevenção de lesão por contenção mecânica**	Realizar técnica correta de contenção mecânica e na indicação certa
	**Prevenção ao suicídio**	Implantar padrão de cuidado específico para pacientes com risco de suicídio:<br>a) **Realizar a avaliação criteriosa do risco:** (1. Identificar fatores de risco 2. Identificar fatores de proteção, 3. Pergunte especificamente sobre suicídio, ideação suicida e história de comportamento suicida).<br>b) **Intervenções Clínicas**: (1. Esforços intensivos para encontrar pacientes que evadem da unidade 2.

**CULTURA DA SEGURANÇA DO PACINTE**		Avaliação dos pacientes que receberam permissão para sair da unidade 3. Os novos pacientes devem estar na maior frequência de observação 5. Avaliação repetida daqueles com maior tempo de permanência devido aos perigos de desespero e cronicidade 6. Fornecimento de medicamentos em dose adequada 7. Recrutamento de apoio familiar antes da alta 8. Prevenção com foco na segurança ambiental 9. Comunicação aprimorada entre equipes 10. Supervisão de pacientes em sofrimento agudo)<br>c) **Reduzir os fatores de risco ambientais**: 1. Eliminar estruturas que são pontos de ancoragem em potencial (drogas, sacolas plásticas) 2. Reduza os dispositivos de estrangulamento 3. Reduza o acesso a objetos perigosos e cortantes 4. Não coloque paciente de risco em leito próximo à janela 5. Elimine cintos, cabides, cortinas de privacidade nas áreas de pacientes de risco 6. Mantenha os dispositivos médicos em área trancada ou sob observação)<br>d) **Implantação de sinalizador de registro de paciente de alto risco**: (1. Estabelecer os sinais de alerta e comunicar à equipe 2. Definir as estratégias internas de enfrentamento em caso de tentativa 3. Utilizar o contato social para distração e apoio 4. Entrar em contato com familiares/amigos para ajudar a resolver uma crise 5. Reduzir acesso do potencial de uso de meios letais).
	**Administração segura de medicamentos**	Envolver o farmacêutico clínico em serviços de reconciliação médica, monitoramento de medicamentos terapêuticos e rondas interdisciplinares de enfermarias.
		Estabelecer uma padronização de medicação e formulários padronizados
		Criar um plano de manejo de medicação, incluindo registro preciso das informações da admissão e conciliar os medicamentos dos pacientes na admissão, transferência e alta.
		Utilizar sistema informatizado de prescrição de medicamentos e gerenciamento de medicações
		Implantar serviço de reconciliação medicamentosa e realizado pelo farmacêutico
		Implementar sistema de abastecimento individual de medicamentos
		Implementar rotulagem da medicação através da técnica "Tall Man Lettering",
		Minimize as distrações durante o processo de administração de medicamentos
	**Administração segura de medicamentos**	Ministrar treinamento de equipe sobre o uso de medicamentos psicotrópicos para incluir usos, efeitos colaterais comuns e monitoramento
		Educação continuada sobre as políticas e procedimentos adequados para administração de medicamentos usando os seis direitos de administração de medicamentos
	**Definição de eventos adversos em psiquiatria**	Incuir, além dos eventos adversos já estabelecidos na cínica, os eventos adversos em psiquiatria: Automutilação intencional ou não intencional; Heteroagressão; Contato sexual; Quedas; Lesões; Eventos Adversos Medicamentosos; Erro de medicação; Ingresso de itens proibidos; Evasão;
		Manter pacientes de risco de heteroagressividade separado dos demais e em local de fácil monitoramento noturno e com quantitativo adequado de equipe

	**Observação de enfermagem noturna**	Utilizar dispositivos para monitorar temperatura, pulso e respiração, traçados de ECG, níveis saturados de oxigênio que possam ser monitorados sem acessar diretamente o paciente dormindo
	**Segurança ambiental**	Proporcionar melhor visibilidade e acessibilidade para os funcionários aos quartos e banheiros dos pacientes
		Fornecer maior visibilidade às portas e corredores de acesso das unidades
		Possibilitar que as unidades de internação ou admissão sejam localizadas em áreas de acesso rápido de uma ambulância
		Estabelecer um protocolo de revista de itens inseguros quanto para pacientes quanto para visitantes em enfermaria psiquiátrica aguda
		Modificar todas as luzes e saídas da unidade para que não possam ser usadas para automutilação
	**Gestão de risco**	Promover admissão de pacientes psiquiátricos de forma planejada e referenciada, oferecendo atendimento psicológico e psiquiátrico para o paciente durante o período de doença aguda, sem interromper o tratatamento psiquiátrico
		Implantar lista de verificação de avaliação de risco psiquiátrico como rotina para enfermeiros estabelecendo a identificação precoce e manejo dessas situações.
		Manter o paciente acompanhado pelo familiar ou cuidador durante o período de internação, preparar o quarto para pacientes de risco; orientar sobre como o paciente deve ser cuidado ao sair do quarto para atividades recreativas, exames ou reabilitação; solicitar à equipe de enfermagem a retirada de medicamentos ou substâncias psicoativas do quarto.
		Destinar um local específico pacientes em estratos de risco de agressão, preparar o espaço físico e alocar pessoal para monitorar pacientes de alto risco.
**Categoria 3**	**Intervenções**	**Ações**
**PLANEJAMENTO DE INTERVENÇÕES**	**Comunicação assertiva**	Reconhecer e ter uma atitude positiva em relação ao papel central da comunicação na segurança do paciente
		Estabelecer transferência fluente de informações através de documentação específica administrativo e de cuidados
		Implantar discussões de equipe na modalidade de debriefing
	**Plano Terapêutico Institucional**	Garantir que a equipe trabalhe em ambientes seguros, bem apoiada, responsável e incentivada a pensar de forma inovadora
		Investir na coleta de dados e análises em tempo hábil
		Ampliar o pensamento sistêmico apropriando da comunicação, aprendizado em equipe, transições, relacionamentos inter e intradepartamentais, integridade operacional e fidelidade

		Investir em treinamento e educação continuada
**Categoria 4**	**Intervenções**	**Ações**
**VIOLÊNCIA INTERPESSOAL**	**Identificação Precoce**	Identificar fatores de risco (delírios persecutórios, dificuldade de comunicação do paciente), melhorar a abordagem do profissional, orientar familiares sobre objetos de risco e importância de não trazer pertences potencialmente perigosos
	**Gestão de Risco**	Reconhecer os fatores preditores de incidente agressivo em internação: falta de moradia, testemunhar abuso, ter um histórico ilegal incluindo uma condenação por agressão, um histórico de perpetrar abuso e ser mais jovem
		Discriminar os pacientes internados entre pacientes agressivos e não agressivos
	**Otimização da equipe**	Introduzir treinamento do conceito de segurança sexual como um direito e como um dever das instituições em garantir ao paciente internado
		Definir o atendimento individualizado em caso de cobertura de pacientes de alto risco de agressão, na proporção de 1 profissional por paciente

*NE: nível de evidência

Referências

[1] Suchting R, Green CE, Glazier SM, Lane SD. A data science approach to predicting patient aggressive events in a psychiatric hospital. Psychiatry Res 2018;268:217–22. https://doi.org/10.1016/J.PSYCHRES.2018.07.004.

[2] Bayramzadeh S. An Assessment of Levels of Safety in Psychiatric Units. HERD 2017;10:66–80. https://doi.org/10.1177/1937586716656002.

[3] Stanley B, Chaudhury SR, Chesin M, Pontoski K, Bush AM, Knox KL, et al. An Emergency Department Intervention and Follow-Up to Reduce Suicide Risk in the VA: Acceptability and Effectiveness. Psychiatr Serv 2016;67:680–3. https://doi.org/10.1176/APPI.PS.201500082.

[4] Short B, Marr C, Wright M. A new paradigm for mental-health quality and safety: are we ready? Australas Psychiatry 2019;27:44–9. https://doi.org/10.1177/1039856218797423.

[5] Steele ML, Talley B, Frith KH. Application of the SEIPS Model to Analyze Medication Safety in a Crisis Residential Center. Arch Psychiatr Nurs 2018;32:7–11. https://doi.org/10.1016/J.APNU.2017.09.005.

[6] Powell-Cope G, Quigley P, Besterman-Dahan K, Smith M, Stewart J, Melillo C, et al. A qualitative understanding of patient falls in inpatient mental health units. J Am Psychiatr Nurses Assoc 2014;20:328–39. https://doi.org/10.1177/1078390314553269.

[7] Kanerva A, Kivinen T, Lammintakanen J. Communication elements supporting patient safety in psychiatric inpatient care. J Psychiatr Ment Health Nurs 2015;22:298–305. https://doi.org/10.1111/JPM.12187.

[8] Marcus SC, Hermann RC, Cullen SW. Defining Patient Safety Events in Inpatient Psychiatry. J Patient Saf 2021;17:E1452–7. https://doi.org/10.1097/PTS.0000000000000520.

[9] Abela-Dimech F, Johnston K, Strudwick G. Development and Pilot Implementation of a Search Protocol to Improve Patient Safety on a Psychiatric Inpatient Unit. Clin Nurse Spec 2017;31:104–14. https://doi.org/10.1097/NUR.0000000000000281.

[10] Camargo ALLS, Neto AM, Colman FT, De Citero VA. Development of psychiatric risk evaluation checklist and routine for nurses in a general hospital: ethnographic qualitative study. Sao Paulo Med J 2014;133:350–7. https://doi.org/10.1590/1516-3180.2013.8100711.

[11] Tavares I de GAM, Peres MA de A, Silva RC da. Adverse events in a psychiatric hospitalization unit. Esc Anna Nery 2022;26:e20210385. https://doi.org/10.1590/2177-9465-EAN-2021-0385EN.

[12] Watts B V., Young-Xu Y, Mills PD, DeRosier JM, Kemp J, Shiner B, et al. Examination of the effectiveness of the Mental Health Environment of Care Checklist in reducing suicide on inpatient mental health units. Arch Gen Psychiatry 2012;69:588–92. https://doi.org/10.1001/ARCHGENPSYCHIATRY.2011.1514.

[13] Tyler N, Wright N, Waring J. Interventions to improve discharge from acute adult mental health inpatient care to the community: Systematic review and narrative synthesis. BMC Health Serv Res 2019;19:1–24. https://doi.org/10.1186/S12913-019-4658-0/TABLES/3.

[14] Okkenhaug A, Tritter JQ, Myklebust TÅ, Deilkås ET, Meirik K, Landstad BJ. Mitigating risk in Norwegian psychiatric care: Identifying triggers of adverse events through Global Trigger Tool for psychiatric care. Int J Risk Saf Med 2019;30:203–16. https://doi.org/10.3233/JRS-190064.

[15] Oliveira A de, Toledo VP. Patient safety in a general hospital's psychiatric hospitalization unit: a phenomenological study. Rev Da Esc Enferm Da USP 2021;55:e03671. https://doi.org/10.1590/S1980-220X2019013103671.

[16] Thibaut B, Dewa LH, Ramtale SC, D'lima D, Adam S, Ashrafian H, et al. Patient safety in inpatient mental health settings: a systematic review. BMJ Open 2019;9:e030230. https://doi.org/10.1136/BMJOPEN-2019-030230.

[17] Kanerva A, Lammintakanen J, Kivinen T. Patient safety in psychiatric inpatient care: a literature review. J Psychiatr Ment Health Nurs 2013;20:541–8. https://doi.org/10.1111/J.1365-2850.2012.01949.X.

[18] Gadzhanova S, Roughead E, Lowy H, O'Connor D. Reducing adverse medication events in mental health: Australian National Survey. Int J Evid Based Healthc 2020;18:108–15. https://doi.org/10.1097/XEB.0000000000000154.

[19] Mills PD, Soncrant C, Gunnar W. Retrospective analysis of reported suicide deaths and attempts on veterans health administration campuses and inpatient units. BMJ Qual Saf 2021;30:567–76. https://doi.org/10.1136/BMJQS-2020-011312.

[20] Reeves E, Henshall C, Hutchinson M, Jackson D. Safety of service users with severe mental illness receiving inpatient care on medical and surgical wards: A systematic review. Int J Ment Health Nurs 2018;27:46–60. https://doi.org/10.1111/INM.12426.

[21] Vantil FCS, Lima E de FA, Figueiredo KC, Massaroni L, Sousa AI, Primo CC. Safety of patients with mental disorders: a collective construction of strategies. Rev Bras Enferm 2020;73:e20170905. https://doi.org/10.1590/0034-7167-2017-0905.

[22] Lee S, Harland KK, Swanson MB, Lawson S, Dahlstrom E, Clemson L, et al. Safety of reassessment-and-release practice for mental health patients boarded in the emergency department. Am J Emerg Med 2018;36:1967–74. https://doi.org/10.1016/J.AJEM.2018.02.026.

[23] De Santis ML, Myrick H, Lamis DA, Pelic CP, Rhue C, York J. Suicide-specific Safety in the Inpatient Psychiatric Unit. Issues Ment Health Nurs 2015;36:190–9. https://doi.org/10.3109/01612840.2014.961625.

[24] Veale D, Ali S, Papageorgiou A, Gournay K. The psychiatric ward environment and nursing observations at night: A qualitative study. J Psychiatr Ment Health Nurs 2020;27:342–51. https://doi.org/10.1111/JPM.12583.

[25] Keers RN, Plácido M, Bennett K, Clayton K, Brown P, Ashcroft DM. What causes medication administration errors in a mental health hospital? A qualitative study with nursing staff. PLoS One 2018;13:e0206233. https://doi.org/10.1371/JOURNAL.PONE.0206233.

[26] Page S, Carr T, Forsyth S, O'Hara A, Burgess J, Charles D. Sexual Safety for In-Patient Mental Health Care-The Democratic Diagnosis of Change. Issues Ment Health Nurs 2019;40:790–7. https://doi.org/10.1080/01612840.2019.1591548.
