# Supplementary material for: Construction of a bundle for the safety of patients with mental disorders during hospitalization
Source: Rev Bras Enferm. 2025 Jan 13;78(1):20230263. doi: 10.1590/0034-7167-2023-0263 (PMC11731854; doi:10.1590/0034-7167-2023-0263)
Supplement: Translation

# Translation: Construção de um *bundle* para a segurança do paciente com transtornos mentais em internação hospitalar

**DOI:** 10.1590/0034-7167-2023-0263pt

**Authors:** Marina Diva de Oliveira, Camila Souza de Almeida, Helen Cristiny Teodoro Couto Ribeiro, Richardson Miranda Machado, Juliano Teixeira Moraes

**Affiliations:** I Universidade Federal de São João del-Rei. Divinópolis, Minas Gerais, Brasil; II Universidade do Estado de Minas Gerais. Divinópolis, Minas Gerais, Brasil

**Keywords:** Segurança do Paciente, Recuperação da Saúde Mental, Transtornos Mentais, Gestão de Riscos, Assistência à, Saúde Mental

## Abstract

**Objetivo:**

construir um *bundle* em segurança do paciente psiquiátrico em internação hospitalar.

**Métodos:**

estudo metodológico realizado em duas etapas. Na primeira, uma revisão abrangente da literatura foi desenvolvida por meio da *scoping review* e conduzida para examinar as evidências sobre a segurança do paciente psiquiátrico em internação hospitalar. Na segunda, com base nas evidências elencadas, foi desenvolvido um conjunto de ações para a segurança do paciente psiquiátrico durante a hospitalização.

**Resultados:**

utilizaram-se 26 artigos publicados no período entre 2012 e 2022, sendo possível categorizar recomendações e construir um *bundle* através de quatro eixos: cultura da segurança; tomada de decisão clínica; planejamento de intervenções; e violência interpessoal.

**Conclusões:**

as evidências científicas forneceram orientações claras sobre as ações para melhoramento da segurança do paciente psiquiátrico durante a internação hospitalar. Essas evidências também destacaram as lacunas existentes na pesquisa, apontando para a necessidade de estudos futuros nessa área.

## INTRODUÇÃO

A prática segura em serviços de saúde constitui um tema de relevância mundial, sendo pauta de importantes avanços nas políticas públicas e práticas de cuidado baseadas em evidência. Para tanto, a avaliação da qualidade dos serviços, a melhoria da segurança do paciente e a cultura de segurança do paciente são componentes estruturais para alcance da efetivação dessa prática^(1)^.

É notório o investimento no gerenciamento de risco e desenvolvimento do cuidado de qualidade e seguro. Contudo, no que se refere à pessoa com transtorno mental, esse investimento não alcança os avanços na mesma proporção testemunhados nos ambientes de outras especialidades. Especificamente, nos serviços de internação hospitalar, identificam-se desafios singulares relacionados ao cuidado seguro dos pacientes psiquiátricos em internação, evidenciando a urgência do investimento em pesquisa, além do desenvolvimento de políticas e aplicação destas para a clínica^(2)^.

O desafio de promover uma assistência segura em saúde mental é ainda mais relevante ao caracterizar a sua especificidade no manejo clínico. Ressalta-se que o paciente com transtornos mentais transita pela Rede de Atenção Psicossocial não apenas em decorrência de demandas relacionadas à ocorrência de eventos e incidentes distintos, como autoagressão, violência e suicídio, mas também de demandas relacionadas às necessidades de cuidados habituais. Tais eventos e incidentes, associados aos demais riscos comuns aos pacientes de outras especialidades, sinalizam a relevância e abrangência deste tema. Portanto, faz-se necessário o convite à ampliação do escopo de estudo quanto à segurança do paciente e discussão acerca do cuidado seguro em paciente com transtornos mentais^(3)^.

A iniciativa da Organização Mundial da Saúde, nomeada como “Direito é Qualidade”, propõe boas práticas em saúde mental que assegurem ao paciente o exercício do seu direto de cidadão. As abordagens propostas transitam pela efetivação da qualidade dos serviços, práticas e segurança do paciente através do foco em *recovery*. O *recovery* representa uma abordagem que objetiva não somente o reestabelecimento da saúde, mas também o fortalecimento e o empoderamento das pessoas que experimentam vivências decorrentes do transtorno mental^(4)^.

Considerando os riscos em que os pacientes psiquiátricos estão vulneráveis e, especificamente, no ambiente hospitalar, estudos apontam maior número de eventos adversos presentes nas internações não psiquiátricas de pacientes com transtorno mental do que naquelas da população em geral. Daumit *et al*.^(5)^ afirmam que, durante as hospitalizações médicas e cirúrgicas, pessoas com esquizofrenia, por exemplo, tinham pelo menos duas vezes mais chances de sofrer eventos adversos graves, quando comparadas com pessoas sem esquizofrenia que sofriam do mesmo problema de saúde. Ainda, segundo os autores, os eventos foram associados aos resultados clínicos ruins durante a hospitalização, o que aumentou o tempo de internação, os custos, o uso de Unidade de Terapia Intensiva e o óbito hospitalar para pacientes com esquizofrenia, alertando para os impactos econômicos e sociais associados ao tema^(6)^.

Sabe-se que um quantitativo significativo de pessoas apresenta algum tipo de transtorno mental. Em nível global, as estimativas indicam que, em todo o mundo, 4,4% das pessoas sofrem de transtorno depressivo e 3,6% de transtorno de ansiedade, apontando para uma tendência sustentada de crescimento desses agravos^(7)^. Além dos efeitos adversos diretos que os transtornos mentais podem produzir, há evidências de que os transtornos mentais estão associados ao aumento na frequência e gravidade de outras doenças crônicas, ao excesso de incapacidades e ao crescimento do absenteísmo no trabalho. Estas evidências explicitam a necessidade de atenção à temática.

Reconhecendo as lacunas de pesquisa em saúde mental e, especificamente, relacionadas à segurança do paciente com transtornos mentais em internação hospitalar, correlacionado aos aspectos de vulnerabilidade apontados, bem como aos avanços e limites do seguimento, este estudo propõe-se na construção de um *bundle* com base nas evidências científicas sobre segurança do paciente com transtornos mentais no contexto hospitalar, partindo-se da seguinte indagação: quais as evidências científicas no contexto da internação hospitalar estão disponíveis sobre segurança do paciente com transtornos mentais?

## OBJETIVO

Construir um *bundle* em segurança dos pacientes com transtornos mentais em internação hospitalar.

## MÉTODOS

### Aspectos éticos

O projeto de pesquisa foi analisado e aprovado pelo Comitê de Ética em Pesquisa, atendendo aos princípios éticos exigidos na Resolução nº 510/2016 do Conselho Nacional de Saúde. O consentimento foi dispensado, por tratar-se de *scoping review* e não contar com uso de materiais biológicos coletados e armazenados como parte das rotinas institucionais, sem adição de risco aos participantes de pesquisas ou prejuízo ao bem-estar dos mesmos.

### Desenho, período e local do estudo

Trata-se de estudo metodológico realizado em duas etapas. A primeira consistiu na realização de *scoping review* para mapear evidências científicas sobre segurança do paciente com transtornos mentais em internação hospitalar. A segunda etapa constituiu em construir um *bundle* a partir da produção do conhecimento mapeada pela revisão. Assim, o *bundle* foi composto de itens sobre segurança do paciente psiquiátrico em internação hospitalar, a partir das evidências recomendadas pelos estudos identificados.

A *scoping review* foi realizada em conformidade com a proposta do JBI, e partiu da seguinte pergunta de pesquisa: quais as evidências científicas no contexto da internação hospitalar estão disponíveis sobre segurança do paciente com transtornos mentais? Este método sugere cinco etapas: 1. Identificação da questão da pesquisa; 2. Identificação dos estudos relevantes; 3. Seleção dos estudos; 4. Análise dos dados; e 5. Agrupamento, síntese e apresentação dos dados^(8,9)^.

### Protocolo do estudo

Foi desenvolvido um protocolo de pesquisa anterior à revisão para dar transparência ao processo. Portanto, esta *scoping review* teve seu protocolo de pesquisa registrado no Open Science Framework, por meio do registro disponível em: https://doi.org/10.17605/OSF.IO/2RMZT .

Para construção da pergunta de pesquisa, utilizou-se a estratégia *Population, Concept* e *Context* (PCC) para uma *scoping review*. A estratégia foi definida da seguinte maneira: P (*Population*) - pacientes com transtornos mentais; C (*Concept*) - segurança do paciente com transtornos mentais; e C (*Context*) - internação hospitalar. Com base nessas definições, foi estabelecida a pergunta norteadora: quais as evidências científicas no contexto da internação hospitalar estão disponíveis sobre segurança do paciente com transtornos mentais?

Para a identificação dos descritores e das palavras-chave, realizou-se uma pesquisa inicial no portal PubMed e na base de dados DeCS/MeSH para identificar os principais descritores e palavras-chave utilizados nos estudos que abordem a temática de interesse a partir da combinação dos MeSH identificados para o mnemônico da pesquisa. Posteriormente, a estratégia de busca definida foi: (*Hospitalization OR Hospitalizations*) *AND* (“*Patient Safety*” *OR* “*Patient Safeties*” *OR* “*Safeties, Patient*” *OR* “*Safety, Patient*”) *AND* (“*Mental Health*” *OR* “*Health, Mental*” *OR* “*Mental Disorders*” *OR* “*Mental Disorder*” *OR* “*Psychiatric Illness*” *OR* “*Psychiatric Illnesses*” *OR* “*Psychiatric Diseases*” *OR* “*Psychiatric Disease*” *OR* “*Mental Illness*” *OR* “*Illness, Mental*” *OR* “*Mental Illnesses*” *OR* “*Psychiatric Disorders*” *OR* “*Psychiatric Disorder*” *OR* “*Behavior Disorders*” *OR* “*Diagnosis, Psychiatric*” *OR* “*Psychiatric Diagnosis*” *OR* “*Mentally Ill Persons*” *OR* “*Mentally Ill Person*” *OR* “*Person, Mentally Ill*” *OR* “*Persons, Mentally Ill*” *OR* “*Mentally Ill*” *OR* “*Ill, Mentally*” *OR* “*Mental Patients*”).

A seleção dos estudos ocorreu entre os meses de julho e setembro de 2022 em sete bases de dados, sendo as seguintes: 1. PubMed/MEDLINE; 2. Literatura Latino-Americana e do Caribe em Ciências da Saúde (LILACS); 3. Embase; 4. Cochrane; 5. CINAHL; 6. *Web of Science*; e 7. Scopus. Além disso, foi realizada busca livre no *Google^®^* e em teses e dissertações sobre a temática, por fornecerem elementos suficientes para compor a revisão e responder à questão de pesquisa.

Foram incluídas pesquisas científicas publicadas na íntegra, em inglês, português e espanhol, até 2021, que versem sobre segurança do paciente com transtornos mentais em internação hospitalar. Excluíram-se protocolos de pesquisas, editoriais, resenhas e cartas. A triagem dos estudos foi realizada utilizando o *Rayyan* ^®^, aplicativo gratuito disponível na *web*, utilizado para auxílio em pesquisas do tipo revisão e metanálise, que permite agilizar a triagem inicial para a leitura de títulos e resumos. Na ocorrência de dúvidas sobre a relevância de estudo a partir do resumo, o artigo completo era recuperado, assim como para todos os restantes estudos que cumpriram os critérios de inclusão. Os estudos excluídos foram aqueles que não estavam alinhados ao objetivo e à questão de revisão estabelecida. A leitura foi realizada por dois revisores independentes, e as divergências foram resolvidas por um terceiro revisor, chegando-se a um consenso.

### Análise dos resultados

Inicialmente, foi realizada a avaliação do título e do resumo de todos os estudos identificados, após foi realizada a leitura completa dos estudos selecionados a partir dos critérios de inclusão descritos. Os dados extraídos dos estudos foram pré-estabelecidos no protocolo de pesquisa. Para o mapeamento, foi utilizado um instrumento estruturado com informações correspondentes ao tipo de estudo, ano de publicação, país de origem, objetivo, tipo de pesquisa, população, local, descrição de cuidados para a segurança dos pacientes com transtornos mentais em internação hospitalar e seus resultados. Tais dados foram inseridos em uma planilha construída no *Microsoft Excel* 2010^®^.

Foi acrescido a esses dados o nível de evidência (NE) metodológica de cada estudo. Para tal, a classificação do nível de evidência dos estudos foi realizada por meio do sistema de classificações preconizados pelo JBI, sendo: nível 5 (opinião de especialistas); nível 4 (estudos observacionais descritivos); nível 3 (estudos observacionais analíticos); nível 2 (estudos quase experimentais); e nível 1 (estudos experimentais). A apresentação dos dados foi de forma descritiva (quantitativo de artigos) com literatura pertinente. Os dados dos estudos foram sintetizados, agrupados e apresentados em quadros.

Na segunda etapa, procedeu-se à construção do *bundle* através das evidências categorizadas na *scoping review*. O *bundle* foi estruturado em quatro categorias, categorizadas pela semelhança do conteúdo, em planejamento de intervenções, cultura da segurança, violência interpessoal e tomada de decisão clínica.

## RESULTADOS

Foram identificados 1.842 estudos; desses, 93 foram exluídos por duplicação. Após triagem inicial dos títulos e resumos, foram inluídos 30 artigos para leitura na íntegra. Após análise, a amostra final foi composta por 26 estudos. A especificidade do tema não pertimitiu encontrar literatura cinzenta que respondesse à pergunta de pesquisa. O processo de seleção dos estudos encontra-se representado no fluxograma *Preferred Reporting Items for Systematic Reviews and Meta-Analyses* (PRISMA) (Figura 1).

**Figura 1:** Fluxograma do processo de seleção dos estudos, 2023

Dos estudos incluídos, 85% (n=22) dos artigos estavam disponíveis em inglês e foram publicados entre 2012 e 2021.

As publicações são advindas de países com maior consolidação de políticas de segurança do paciente, como é o caso dos EUA, e de saúde mental, como é o caso do Brasil, e representam locais em que práticas inovadoras de cuidado estão sendo estabelecidas.

O Brasil está em 3º no *ranking* de publicações, e o único na América Latina com publicação nesta área. As publicações brasileiras versaram nas áreas sobre avaliação de risco psiquiátrico, eventos adversos em internação psiquiátrica, compreensão da segurança do paciente pela equipe de hospital geral e implantação de gerenciamento de risco para segurança do paciente.

Diante da amplitude de temas abordados nas publicações encontradas, foi necessário estratificar os resultados em categorias temáticas. Analisando os achados e resultados mais relevantes dos estudos, realizou-se a categorização por grandes áreas temáticas e subcategorias, como cultura da segurança, tomada de decisão clínica, planejamento de intervenções e violência interpessoal, apresentadas no Quadro 1.

**Quadro 1:** Categorização dos temas abordados nos artigos incluídos no estudo. Minas Gerais, Brasil, 2023

Categoria	Subcategorias
Cultura da segurança	Erro de medicação; práticas de segurança na administração de medicamentos; melhoria da qualidade; segurança sexual; gestão de risco; eventos adversos; observações de enfermagem noturna; segurança do ambiente físico; comunicação assertiva e ativa; risco de queda; comportamento nocivo: automutilação, comportamento suicida e autonegligência (A2, A4, A5, A6, A7, A8, A9, A10, A11, A14, A15, A18, A19, A21, A23, A24, A25, A26)^(3,7,10-25)^
Tomada de decisão clínica	Cuidados continuados após alta; planejamento para alta; cuidado planejado em internação de pacientes com transtornos mentais graves; *checklist* de avaliação de risco psiquiátrico e rotina (A13, A20, A22)^(26-28)^
Planejamento de intervenções	Micropolítica dos cuidados em pacientes psiquiátricos em internação (A3, A16, A17)^(2,29-31)^
Violência interpessoal	Abordagem relacionada à preocupação quanto aos episódios de violência do paciente e equipe (A1, A12)^(31,32)^

A categoria “cultura da segurança” representou 69% (n=18) das publicações, com variedade de subcategorização, as quais correlacionam com os principais eixos temáticos tratados nas políticas e diretrizes em “segurança do paciente”. A categoria referente à “tomada de decisão clínica” representou 12% das publicações (n=3), bem como a categoria “planejamento de intervenção” (n=3). A categoria “violência interpessoal” representou 8% das publicações (n=2) (Quadro 1).

Através de tais evidências, foi elaborado o *bundle* em segurança do paciente psiquiátrico em internação hospitalar (material suplementar).

Em virtude dos achados e da complexidade dos eixos, foi elaborado um *bundle* multiaxial que alinha um conjunto de *bundle* em correspondência às categorias e suas respectivas intervenções. Foi estabelecido através de quatro eixos: cultura da segurança; tomada de decisão clínica; planejamento de intervenções; e violência interpessoal. Todos estavam alinhados aos estândares internacionais da abordagem em *recovery* através da segurança e qualidade como direitos humanos.

## DISCUSSÃO

Conforme observado, as publicações abordaram estratégias que podem ser consideradas de relevância para o cuidado seguro ao paciente psiquiátrico em internação. Dessa forma, foi construído um *bundle* com as principais recomendações sobre segurança do paciente psiquiátrico em internação hospitalar. As temáticas abordadas partiram do viés da produção do cuidado e das estratégias com foco em direito como qualidade.

As evidências estratificadas por categorias possibilitaram elencar recomendações como: avaliar o paciente; realizar a identificação do risco; criar uma cultura organizacional e estrutural; conscientizar os colaboradores; organizar o processo de trabalho tanto na internação quanto na alta referenciada e segura; identificar os sinais de piora e gatilhos de agravamentos; administrar seguramente medicação; minimizar os riscos de queda; prevenir o suicídio e episódio de heteroagressividade.

Os estudos categorizados na cultura de segurança do paciente mencionam sobre os processos investigados de tratamento e cuidado em internação de pacientes com transtornos mentais.

Dentro da cultura de segurança do paciente, alguns seguimentos devem ser pontuados e compreendidos para elaboração de estratégias que fortaleçam a política de segurança do paciente do paciente psiquiátrico dentro das instituições de saúde.

Quanto aos eventos adversos em internação psiquiátrica, incialmente, é importante compreender que os eventos adversos são ao menos, parcialmente, resultado da omissão ou prestação de cuidados clínicos. Podem resultar de doenças psiquiátricas e são influenciados por muitos fatores do paciente, clínicos e sociais. Os pacientes psiquiátricos possuem elevado de risco de sofrer eventos adversos, levando em consideração a associação entre as especificidades terapêuticas e a sintomatologia das psicopatologias que, por vezes, levam o paciente a comportamentos como agressividade, isolamento, suicídio, fuga e autodefesa reduzida^(10,23)^.

Destacam-se a tomada de decisão na prática clínica, como o exercício da atuação baseada na extração de evidências, interpretação e gerenciamento dos riscos, integração de um plano de cuidado junto ao paciente, além de foco no processo saúde-doença e revisão das ações e resultados para aumentar a condição de saúde e recuperação do paciente. Além disso, o desenvolvimento de julgamentos e decisões clínicas relacionadas ao gerenciamento de incidentes, avaliação de risco, diagnóstico e a gestão da alta são decisivos foram temas de publicação nos estudos analisados.

Nesta categoria, a gestão da alta hospitalar é uma etapa importante a ser considerada para o cuidado ao paciente psiquiátrico, visto a relação com a ocorrência de reinternações e a ligação dos transtornos mentais e doenças crônicas. Trata-se de processo iniciado na internação, para que seja realizado o mapeamento do paciente em seu contexto integral para identificação de vulnerabilidades e fragilidades. A partir disso, construiu-se uma rede de cuidado em seu território. Portanto, reconhece-se que, para uma alta segura, os recursos e planejamento do cuidado devem transitar em todo o período da internação, além de envolver os cuidados pós-alta hospitalar, assegurando o tratamento e a recuperação de forma humanizada^(33)^.

No processo de gestão da alta, houve destaque ao papel do enfermeiro. O conhecimento sobre os documentos da alta, a orientação sobre a continuidade do tratamento de forma clara e eficaz, o reconhecimento das possíveis limitações clínicas e da compreensão do paciente quanto ao tratamento, quadro clínico e sinais de piora, e o envolvimento da família/responsável e comunidade devem acontecer e sanar todas as dúvidas na alta hospitalar^(33)^. Esse processo pode influenciar a redução de readmissão, melhoria do bem-estar, aumento da adesão ao tratamento, principalmente destacando a relação com a redução ao suicídio.

Os estudos relacionaram, principalmente, a prevalência, a gestão e a prevenção de comportamentos violentos/agressivos. A agressão do paciente em ambientes de saúde mental é um risco de maior relevância relacionado aos pacientes psiquiátricos, em virtude da sintomatologia de alguns transtornos mentais. Apresenta-se como um desafio contínuo para as organizações e profissionais de saúde, visto a peculiaridade do manejo, bem como a ocorrência de trauma físico e psicológico para outros pacientes, funcionários e visitantes^(31)^.

Neste sentido, destaca-se que sinais agressivos podem reduzir a agressão, ou seja, há possibilidade de prevenção da agressão através da antecipação da violência, se ocorrer a partir de manejos clínicos correspondentes aos riscos identificados.

O cuidado seguro advém também da assistência e cultura da segurança, consolidados pelas atitudes, comportamentos e valores dos profissionais e das organizações. Ao desenvolver a segurança do paciente, é importante lembrar a diversidade interrelacional do conceito para que todas as áreas sejam consideradas no trabalho de desenvolvimento^(30)^. É necessário investimento em pesquisa, desenvolvimento de políticas e tradução destas para prática clínica.

A consolidação de estratégias que considerem a realidade local dos serviços, identificando as fragilidades organizacionais e, também, dos profissionais, possibilita elaborar macro e microintervenções mais assertivas.

A micropolítica do trabalho em saúde mental foi apresentada como um espaço de envolvimento com a singularidade e fragilidades dos pacientes, reconhecendo os desafios extrínsecos e intrínsecos e a complexidade do cuidado de pacientes psiquiátricos em internação. Pressupõe-se que a segurança do paciente se dará a partir da organização e exercício de práticas conjuntas.

Revela um trabalho estabelecido com o encontro entre a produção do cuidado, a gestão e a formação, proposto como um processo analítico e problematizador das práticas estabelecidas e das linhas de conexão

Os resultados demonstram iniciativas para um cuidado seguro em pacientes psiquiátricos, porém de forma tímida. Sabe-se que existe uma negligência histórica com relação à saúde mental.

Embora as 26 evidências encontradas apoiem a eficácia de muitas intervenções em segurança do paciente psiquiátrico em ambiente hospitalar, há uma notável insuficiência de pesquisas que direcionem questões amplas de cunho cultural, social e econômico e que, verdadeiramente, afetem o cuidado seguro como uma prática de *recovery* e intervenções pontuais que possam abordá-las.

Portanto, este estudo reforça a existência de lacunas em pesquisas na saúde mental em segurança do paciente psiquiátrico em internação hospitalar.

Contudo, os estudos apresentados direcionam um cuidado seguro através de intervenções baseadas em evidências que se mostraram eficazes. Podem ser reconhecidos como um *menu* de opções de ações ancoradas na segurança do paciente nos serviços hospitalares, revelando caminhos possíveis para a melhoria contínua e efetiva consolidação da cultura da qualidade. Dessa forma, este *bundle* em segurança do paciente com transtornos mentais em internação hospitalar, como uma ferramenta de cuidado sistematizado, valida a importância do pensamento crítico sobre o cuidado seguro necessário, principalmente em um ambiente de alta vulnerabilidade.

Ainda que existam desafios para realizar essa abordagem, os exemplos de evidências aqui apresentados e listados, através das recomendações do *bundle*, revelam concretamente o que pode ser feito e em quais áreas de pesquisas devemos investir.

### Limitações do estudo

Embora a pesquisa tenha possibilitado gerar conhecimento, muitos estudos que podem abordar os cuidados com a segurança do paciente nem sempre usam este termo em seus descritores e, portanto, podem não ter sido incluídos no estudo.

### Contribuições para as áreas da enfermagem, saúde ou políticas públicas

No âmbito das políticas públicas de segurança do paciente, embora tenham ocorrido avanços significativos, ainda há muito trabalho a ser feito, e a enfermagem desempenha um papel de destaque nessa área. Assim, este estudo contribui para a conexão entre conhecimento e prática, com o objetivo de possibilitar à enfermagem entrelaçar saberes e práticas em um cuidado seguro, humano e ético na assistência à saúde mental.

## CONCLUSÕES

Com base nas recomendações evidenciadas pela *scoping review*, foi possível a elaboração um *bundle* para assegurar a segurança dos pacientes psiquiátricos no ambiente hospitalar.

Todas as publicações versam sobre um cuidado projetado com governança para prevenção de riscos e consolidação da segurança do paciente psiquiátrico em internação. A *scoping review* informa sobre as evidências no campo da segurança do paciente psiquiátrico no ambiente da internação, atendendo à pergunta de pesquisa.

No *bundle*, foi possível categorizar quatro eixos que apresentam o foco da assistência: cultura da segurança; tomada de decisão clínica; planejamento de intervenções; e violência interpessoal. Todos foram alinhados aos estândares internacionais da abordagem em *recovery* através da segurança e qualidade como direitos humanos.

Este instrumento, construído através de uma síntese das evidências, poderá contribuir para as futuras pesquisas, programas e políticas, por proporcionar ao pesquisador um consenso quanto às recomendações de cuidado seguro identificados pelas publicações atuais. Além disso, podem-se identificar as prioridades de pesquisa relacionadas à temática.

Por fim, evidenciaram-se a complexidade do cuidado em saúde mental e a oportunidade para realçar a relevância da segurança do paciente em internação na consolidação do cuidado com foco em *recovery*.

## Data Availability

https://doi.org/10.17632/nvn7byzf7z.1
